# Revolutionizing hysteroscopy outcomes: AI-powered uterine myoma diagnosis algorithm shortens operation time and reduces blood loss

**DOI:** 10.3389/fonc.2023.1325179

**Published:** 2023-12-08

**Authors:** Minghuang Chen, Weiya Kong, Bin Li, Zongmei Tian, Cong Yin, Meng Zhang, Haixia Pan, Wenpei Bai

**Affiliations:** ^1^ Department of Obstetrics and Gynecology, Beijing Shijitan Hospital, Capital Medical University, Beijing, China; ^2^ Department of Magnetic Resonance Imaging (MRI), Beijing Shijitan Hospital, Capital Medical University, Beijing, China; ^3^ Information Center, Beijing Shijitan Hospital, Capital Medical University, Beijing, China; ^4^ College of Software, Beihang University, Beijing, China

**Keywords:** artificial intelligence (AI), submucosal myomas, instance segmentation, hysteroscopic myomectomy, magnetic resonance imaging (MRI)

## Abstract

**Background:**

The application of artificial intelligence (AI) powered algorithm in clinical decision-making is globally popular among clinicians and medical scientists. In this research endeavor, we harnessed the capabilities of AI to enhance the precision of hysteroscopic myomectomy procedures.

**Methods:**

Our multidisciplinary team developed a comprehensive suite of algorithms, rooted in deep learning technology, addressing myomas segmentation tasks. We assembled a cohort comprising 56 patients diagnosed with submucosal myomas, each of whom underwent magnetic resonance imaging (MRI) examinations. Subsequently, half of the participants were randomly designated to undergo AI-augmented procedures. Our AI system exhibited remarkable proficiency in elucidating the precise spatial localization of submucosal myomas.

**Results:**

The results of our study showcased a statistically significant reduction in both operative duration (41.32 ± 17.83 minutes vs. 32.11 ± 11.86 minutes, *p*=0.03) and intraoperative blood loss (10.00 (6.25-15.00) ml vs. 10.00 (5.00-15.00) ml, *p*=0.04) in procedures assisted by AI.

**Conclusion:**

This work stands as a pioneering achievement, marking the inaugural deployment of an AI-powered diagnostic model in the domain of hysteroscopic surgery. Consequently, our findings substantiate the potential of AI-driven interventions within the field of gynecological surgery.

## 1 Introduction

Uterine fibroids, also known as myomas, are the most common benign tumors affecting the female reproductive system. They are most prevalent among patients aged 30-50 years (1). By the age of 50, the cumulative incidence of myomas in women can reach up to 70%—80%. Among all myomas, submucosal myomas account for approximately 5.5%-10% (2). Submucosal myomas often lead to abnormal uterine bleeding, infertility, recurrent pregnancy loss, and pelvic pain (3).

Submucosal myomas were divided into three subtypes. In 1993, Wamsteker et al. introduced a classification system for submucosal myomas based on the degree of intramural extension during hysteroscopic myomectomy (4). According to this system, submucosal myomas are categorized as type 0, 1, or 2. This classification system was adopted by the European Society of Hysteroscopy and served as the basis for the myoma subclassification system established by the International Federation of Gynecology and Obstetrics (FIGO) (5). Hysteroscopic myomectomy is considered the optimal method for type 0 myomectomy, and the slicing technique is now widely accepted as the standard approach (6). However, the International Society for Gynecologic Endoscopy (ISGE) suggests intrauterine morcellation (IUM) as an alternative option due to its advantages in terms of learning curve and operative time (7, 8).

However, the FIGO subclassification system also has clinical limitations. Uterine myomas will usually distort uterine structure. It will increase the difficulty to distinguish the extent of myometrial invasion, reducing the accuracy of FIGO system (9). Magnetic resonance imaging (MRI) has become an essential imaging modality for assessing myomas. In the clinical management of myomas, MRI T2-weighted images, particularly sagittal images, are commonly utilized. MRI has demonstrated significant advantages in terms of accurately determining the number, size, and location of uterine myomas (10). Conventional MRI can clearly differentiate from uterine myomas, myometrium, and endometrial layers. Myomas exhibit low signal intensity in T2-weighted images, allowing for a clear demarcation of their boundaries from the surrounding myometrium. Study by Wilde, S. and S. Scott-Barrett has indicated that MRI exhibits higher sensitivity in detecting small submucosal myomas measuring less than 5 mm (11). And MRI images were obtained in a more objective way, not dependent on the operator’s experience.

Traditional imaging diagnosis heavily relies on the expertise and experience of physicians. However, artificial intelligence (AI), based on machine learning and deep learning techniques, offers significant advantages in terms of image feature extraction, repeatability, and objectivity, thus aiding in decision-making process (12).

The approaches powered by AI can enhance the efficiency and reliability of diagnoses, ultimately benefiting patient care and outcomes. AI has demonstrated promising results in the diagnosis of breast cancer (13), prostate cancer (14), and brain glioma (15) using MRI. However, its application in the diagnosis of gynecological tumors is still in its early stage. Robin Wang et al. conducted a study that they utilized AI and MRI data to differentiate between benign and malignant ovarian tumors (16). They developed a deep learning model that outperformed primary radiologists in terms of accuracy and specificity. Moreover, the AI model enhanced the specificity of diagnosis for both junior and senior radiologists. Tang et al. proposed a new segmentation network using deep learning based on T2-weighted sagittal MRI data, exhibiting high sensitivity and specificity in the diagnosis of uterine diseases (17). They modified a convolutional neural network to achieve automatic segmentation of uterine MRI images based on T2-weighted signatures, covering uterine endometrial cancer, uterine cervical cancer, and uterine myomas. This approach demonstrated the feasibility of diagnosing uterine myomas through a deep learning approach (17, 18). But the existing studies mostly focused on the semantic segmentation, limiting the identification among the myomas, uterine wall and uterine cavity.

Our team has successfully constructed a large-scale uterine myoma MRI dataset that covers all FIGO types. This dataset comprises a substantial number of T2-weighted sagittal images of myomas, along with corresponding annotation files. Additionally, we have developed an instance MRI segmentation model based on deep learning, which significantly contributes to myoma classification and facilitates surgical decision-making through precise instance segmentation of myomas, uterine wall, and cavity (19).

This research represents the first endeavor to introduce AI technology into the realm of operative decision-making for submucosal myomas. By utilizing AI model based on MRI, surgeons could be better prepared. Consequently, patients can benefit from various advantages, such as less bleeding and shorter operation duration, highlighting the potential of AI applications in hysteroscopic myomectomy.

## 2 Methods

### 2.1 Participants and study design

Participants in this study were enrolled from January 2022 to January 2023 at Beijing Shijitan Hospital. 56 patients were included with age ranging form 39 to 46 years old and in size 2.43± 0.77 cm. This study was conducted in accordance with the World Medical Association’s Declaration of Helsinki. And it was approved by the scientific research ethics committee of Beijing Shijitan Hospital, Capital Medical University (code: sjtkyll-lx-2022 (1)). This study would not violate the rights and interests of patients. The ethics committee clearly stated that specific consent procedures were not required for this study.

Participants met the followed inclusion criteria: 1) with symptoms such as abnormal uterine bleeding, infertility, and recurrent pregnancy loss; 2) diagnosed with submucosal myomas by magnetic resonance imaging (MRI); 3) The postoperative pathology was submucosal myoma. The exclusion criteria were as follows: 1) with severe comorbidities; 2) acute stage of pelvic inflammation and vaginal inflammation, or body temperature >37.5°C; 3) uterine active massive bleeding, severe anemia; 4) normal pregnancy status; 5) history of uterine perforation within 3 months; 6) invasive cervical cancer; 7) genital tuberculosis without anti-tuberculosis treatment; 8) with MRI contraindications, such as febrile convulsions, active foreign bodies in the eyes, cardiac pacemakers, metal intrauterine devices, metal joints and metal dentures; 9) postoperative pathology excluded uterine myomas.

All eligible subjects underwent MRI examination. All eligible subjects were equally divided into 2 groups with the method of random number table. Half of them were divided into group MRI-AI, and the other half were divided into group MR. Bipolar electrosurgical system was employed and intrauterine pressure was set at 100 mmHg during hysteroscopic myomectomy in this study. The surgical procedure in both groups was performed by the same surgeon with abundant experience.

### 2.2 MRI image acquisition

MRI examination in this study was completed in the PHILIPS INGENIA magnetic resonance imaging system with 3.0T ultra-high field. The MRI scan parameters were as follows: repetition time 4200ms, echo time 130ms, voxel 0.8x0.8x4.0cm3, field of view 24x24cm, reverse angle 90°. MRI provided multiple images from the sagittal, coronal and axial scans and from various sequences including T1W, T2W, mDIXON and DWI. The image resolution was larger than 512x512 pixel. T2W sagittal images were finally collected for the followed image processing.

### 2.3 MRI image instance segmentation

MRI image was processed based on the instance segmentation model which has been published by our team (19).

MRI images are characterized by the presence of offset fields, low contrast and blurred uterine tissue boundaries, which increasing difficulty in AI automatic segmentation. In order to solve this problem, adaptive histogram equalization was used to adjust the contrast between uterine tissues, especially for uterine myomas and uterine wall with slightly similar signal intensity. The N4ITK method was used to correct the offset field problem, and the Z-Score method was used to normalize the MRI images to the same range.

A specialized network architecture was designed for image processing. Firstly, HRNetv2p was used for high-resolution feature extraction and multi-scale feature fusion operations in the backbone section, so that small scale targets in the uterine region can also be extracted effectively. And DCN was used to address the issue of diverse organ shapes, to extract true feature information from different shapes, and to reduce the loss of shape information. CBAM modules were used to assist in feature extraction, filter out irrelevant and interfering feature information, and enhance the feature expression ability of the AI model. An anchor-based approach was used to assist in target localization.

The size of the myoma, uterine wall and uterine cavity within the uterine region varies considerably so that conventional size settings are no longer suitable. Our previous work conducted distribution statistics on the length, width, and aspect ratio of the target minimum peripheral bounding box on our dataset, providing reference for MR image processing. K-Means clustering method was used to calculate the number of clusters in the target bounding box and output the box size. Simultaneously, it is applied to different feature layers for better detection of small-scale targets in the shallow layer and large-scale targets in the deep layer. Finally, the PointRend module was introduced in the segmentation task to continuously optimize the segmentation edges between adjacent targets using an iterative segmentation strategy. This algorithm reduced jaggies and rough edges, resulting in smoother and more detailed edges for various objects within the uterine region. Since the model contains multiple subtasks, the loss function also consists of multiple parts. The classification loss function tested the accuracy of target classification by using cross entropy loss. The bounding box loss function detected the accuracy of target localization by using smooth L1. The segmentation loss function consists of two parts, CoarseMaskHead and MaskPointRend, which are mainly calculated by binary cross-entropy loss.

### 2.4 Measurement methods

The clinical data, including age, weight, height, pregnancy times, abortion times, clinical symptoms, preoperative hemoglobin value, operation time, bleeding, and fluid deficit, were analyzed in this study. The size, type and position of submucosal myomas were measured using MRI and AI models we built. And the final hysteroscopic myomectomy was the gold standard according to the FIGO system. A graduated plastic bag was placed under the participant to collect the effluent fluid during operation, contributing to get the fluid deficit. Cervical dilatation time was not included in the operation time.

### 2.5 Statistical analysis

Statistical analysis was realized using the SPSS software (version 29.0, SPSS Inc., Chicago, IL, USA). Quantitative data that conform to normal distribution were expressed as mean ± standard deviation (SD). Comparisons between the data were performed with *t* test. Quantitative data that do not fit a normal distribution are expressed as percentiles. Comparisons between the data were performed with Mann-Whitney U test. Qualitative data were expressed as number and percentage. And chi-square test was performed to analyze the difference of the two groups. Probability values of *p*<0.05 were considered significant.

## 3 Results

### 3.1 General clinical characteristics

The clinical characteristics were similar in both groups MRI and MRI-AI. No significant differences in terms of age, height, weight, BMI, time of pregnance, and the level of hormone (*p*>0.05, **Table 1**) were found. The symptoms, such as abnormal menstruation and anaemia, caused by myoma were also similar in the two groups(*p*>0.05). Besides, there are no significant differences in the size (2.44 ± 0.64cm vs. 2.43 ± 0.90cm, *p*=0.99) and type ((type 0:10[35.71%] vs. 8[28.57%], *p*=0.39; type 1: 13[46.43%] vs. 14[50.00%], *p*=0.50; type 2: 5[17.86%] vs.6[21.43%], *p*=0.50) of each myoma between group MRI and group MRI-AI.

**Table 1:** General clinical characteristics.

	Total	MRI	MRI-AI	*p*-value
patients(n)	56	28	28	–
Age	43(39-46)	43.50(39.25-47)	42(38.25-45)	0.13
height(cm)	161.43 ± 4.89	160.93 ± 5.29	161.93 ± 4.49	0.45
weight(kg)	62(56-67)	62.50(55.25-71.75)	62.00(56.25-66.50)	0.94
BMI(kg/m^2^)	23.73(21.47-26.16)	23.97(21.10-26.48)	23.20(21.54-25.82)	0.86
pregnance	2(1-3)	2(1-3)	2(1-3)	0.72
vaginal delivery	1(0-1)	1(0-1)	1(0-1)	0.29
cesarean section	0(0-0)	0(0-0)	0(0-0.75)	0.81
aborion	1(0-2)	1(0-2)	1(0-2)	0.84
prolonged menstruation(n[%])	22[39.29]	10[35.71]	12[42.86]	0.55
menorrhagia(n[%])	31[55.36]	16[57.14]	15[53.57]	0.50
anaemia(n[%])	10[17.86]	4[14.29]	6[21.43]	0.36
giddy(n[%])	20[35.71]	8[28.57]	12[42.86]	0.20
HGB(g/l)	121.50(93.75-130.50)	122.50(97.00-130.00)	122.00(84.00-130.50)	0.56
myoma size(cm)	2.43 ± 0.77	2.44 ± 0.64	2.43 ± 0.90	0.99
type-0(n[%])	18[32.14]	10[35.71]	8[28.57]	0.39
type-1(n[%])	27[48.21]	13[46.43]	14[50.00]	0.50
type-2(n[%])	11[19.64]	5[17.86]	6[21.43]	0.50
progesterone(ng/ml)	0.88(0.31-4.56)	0.58(0.31-5.48)	1.23(0.31-2.74)	0.90
estrogen(pg/ml)	83.33(57.75-156.06)	78.79(30.21-175.32)	93.06(63.67-135.85)	0.59

### 3.2 MRI image instance segmentation

**Figure 1:**
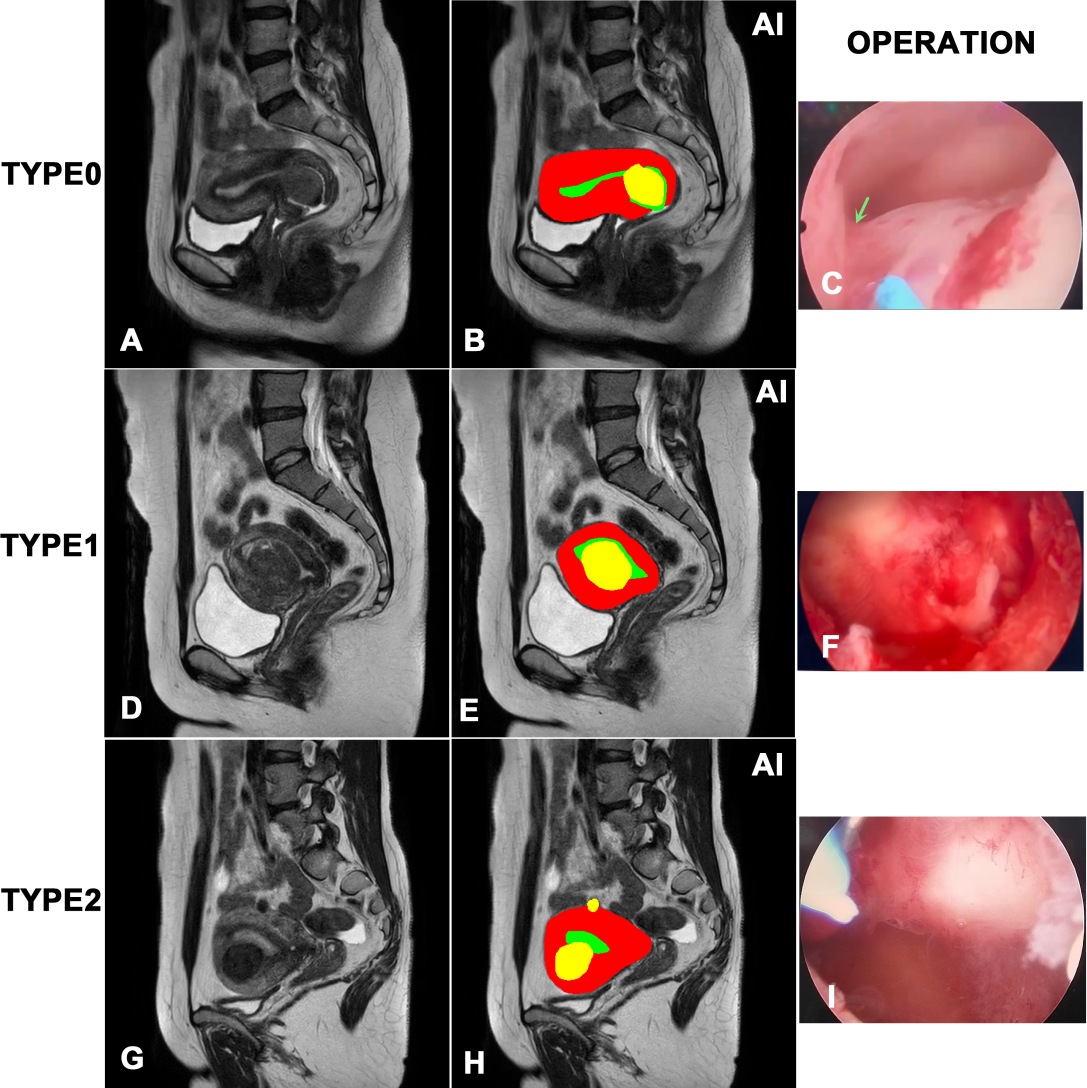
Visualization of the instance segmentation of our AI model. The left side represents original MRI image, and the right side represents the inference masks generated by our AI model. In the masks of model inference, yellow represents myomas, red represents the uterine wall, green represents the uterine cavity. **(A, B)** and **(C)** represents type 0 myoma. **(D, E)** and **(F)** represents type 1 myoma. **(G, H)** and **(I)** represents type 2 myoma.

**Figure 1** showed the results of the instance segmentation of AI model. MRI images of submucosal myomas, including type 0, type 1 and type 2, were segmented by AI model. Inference masks were covered on the original MRI images, representing myomas, uterine cavity and uterine wall. The left side represents original MRI image, and the middle side represents the inference masks generated by our AI model. And the right side represents the intraoperative view. The inference masks clearly showed the position of myomas, uterine cavity and uterine wall in relation to each other. And it was verified by the intraoperative view. A, B and C showed that the myoma is attached to the lower part of the uterine cavity by a stalk(green narrow), representing the type 0 myoma. D, E and F showed that the intrauterine penetration of myoma exceeded 1/2, which represented it is type 1 myoma. G, H and I showed the bigger myoma is <50% submucosal and≥50% intramural, representing the type 2 myoma.

### 3.3 Myoma type matching and perioperative data

**Table 2** showed the myoma type consistency and perioperative data in group MRI and group MRI-AI. The type consistency rate in group MRI-AI was higher than that in group MRI, but not statistically significant (24[85.71] vs. 26[92.86], *p*=0.34). Although no significant differences in terms of fluid in, fluid out and fluid deficit were found(*p*>0.05), the difference in operation time (41.32 ± 17.83min vs. 32.11 ± 11.86min, *p*=0.03) and bleeding (10.00(6.25-15.00) ml vs. 10.00(5.00-15.00) ml, *p*=0.04) was reported to be statistically significant.

**Table 2:** Type matching and Perioperative data.

	Total	MRI	MRI-AI	*p*-value
type consistency(n[%])	50[89.29]	24[85.71]	26[92.86]	0.34
operation time(min)	36.71 ± 15.70	41.32 ± 17.83	32.11 ± 11.86	0.03
fluid in(ml)	2000.00(1000.00-3000.00)	2250.00(1500.00-3000.00)	2000.00(1000.00-2750.00)	0.16
fluid out(ml)	1600.00(762.50-2500.00)	1850.00(1100.00-2650.00)	1400.00(712.50-2325.00)	0.11
fluid deficit(ml)	392.86 ± 179.76	408.93 ± 182.09	376.79 ± 179.24	0.51
bleeding(ml)	10.00(5.00-15.00)	10.00(6.25-15.00)	10.00(5.00-15.00)	0.04

## 4 Discussion

The application of AI powered algorithm in clinical decision-making is globally popular among clinicians and medical scientists (20). Artificial intelligence, especially machine learning, reinforcing repetition learning and deep learning, are particularly well-suited to deal with challenges in healthcare industry. Decision trees are common tools used by clinicians, which aim to make accurate decision in daily practice (21). Convolution neural networks, role as a deep learning model, aim to decipher various clinic images to help make diagnosis (22). Some machine learning models that can risk-stratify patients in preparation for surgery will help clinicians identify high-risk factors and optimize the healthcare progress (23, 24). There are still many challenges. The adoption of AI has the potential of making unsound decision and inadvertent bias, which means the training curricula renewal and the update of knowledge are necessary (25). Moreover, the black-box process which most AI models present make it hard for clinicians to trust and explain to the patients, which restrict their application in healthcare practice. In this article, to make it more acceptable in practice, we explain our AI algorithm in detail rather than simply give out the figure and data. We are willing to construct an accurate, locally calibrated and clinically accessible AI powered algorithm to risk-stratify patients with submucosal uterine myoma and to optimize patients’ care process.

Uterine myomas, occurring in 70% of women in their reproductive years, are the most common benign, solid, pelvic tumors in women. The minimally invasive operations choice for uterine myomas includes laparoscopy and hysteroscopy, which is the common indication for the submucosal myomas. A meta-analysis showed that, compared with infertile women without submucosal myomas, those who are infertile women with submucosal myomas showed significantly lower pregnancy and delivery rates (26). Consequently, the resection of submucosal myomas led to a significant increase in the pregnancy rate. Research also indicates that the operation effectively decrease the patients’ blood loss (27). Submucosal myomas, which grouped as type0,1 and 2, are almost removable only with hysteroscopy, which means the preoperative recognition and classification of myoma is meaningful and decisive in clinical practice.

In a retrospective cohort study designed by Mayo Clinic, preoperative MRI FIGO myoma staging read by experts is not completely consistent with surgical description, the variation of which is clinically significant. The author concluded that additional validation of FIGO staging is needed, however, in the other way, it means that even experts’ ability to map the lesion is not always stable and liable (9).

However, only preoperative myoma mapping is not enough. The image character also make difference. In a prospective study, according to the signal intensity of T2-weighted MR images, myomas are classified into 3 types: low intensity; intermediate intensity; high intensity, which kinds of classification method is totally different from the method we mentioned before which is classified by the positional relationship between uterine myoma and myometrium (28). In conclusion, the researchers assumed that the myomas with the high intensity in T2-weighted MR images should be exempted from a kind of specific myoma surgery, the magnetic resonance-guided focused ultrasound surgery, because the postoperative outcome is unfavorable. It hints us that not only the positional relationship between uterine myoma and myometrium is meaningful, the MR signal intensity also makes the difference, which our AI algorithm will take into account.

The time of operation influence the preoperative recovery, as well as the loss of blood. Our data firmly proved that the AI algorithm group experienced shorter operation time and less loss of blood. The concept of prehabilitation or enhanced recovery after surgery (ERAS) is frequently mentioned and quoted by the clinicians, which aim to speed up the progress of postoperative recovery. The AI algorithm can also help to make the patients who experienced hysteroscopy recover faster, because its application shortens the operation time. In a prospective randomized controlled trail, researchers reported that intracervical vasopressin injection during hysteroscopy reduces intraoperative bleeding (29). In another RCT study, oxytocin drip during hysteroscopy showed the similar effect. However, hypotension, arrhythmia, hyponatremia, might occur after administration of oxytocin and vasopressin, which seriously restrict the medication administration. The AI algorithm help to showed the same result but not lead to any side effect.

In addition to fluid overload and hyponatremia, hysteroscopy is associated with several other immediate complications, including uterine perforation, air embolism, transient blood oxygen desaturation, hypercapnia, and coagulopathy (29–31). However, it is noteworthy that none of these complications were observed among the participants in our study. The absence of these complications could be attributed, in part, to the relatively modest sample size of our study.

Our prior research introduced a deep learning-based instance segmentation model capable of automatically generating output encompassing the class, location, and masks related to the uterine wall, uterine cavity, and uterine myomas (19). Although MRI has been proved to be a useful technique to diagnose uterine myoma and enable clinicians to select appropriate management (32, 33), there are few similar studies with limited transformation and application. In 2017, Korean researchers proposed a 3D reconstruction method with uterine MRI templates enables 3D visualization of myomas (34). This article exclusively focuses on the methodology aspect, without delving into real-world applications or providing a comparison with operative foundations. As a result, its practical applicability might be viewed with skepticism. Furthermore, three dimensional printed uterine model from MRI was constructed to guide the operation and choose the uterine incision in a pregnancy woman, at the same time with caesarean section (35), which is just a case report with restricted clinical evidence. The author asserts that the expense and complexity associated with producing the three-dimensional model were comparatively modest. This emphasizes the attainability of employing 3D models within the realm of myoma operations. In another research, a 3D MRI model was drawn preoperatively and taken into application in clinical practice (36). The deduction that the present study surpasses conventional 2-dimensional MRI in accurately identifying the locations of uterine myomas and endometrium was based on a web-based survey distributed to gynecologists. However, this conclusion lacks precise data on factors such as blood loss or operation time. Based on our current accomplishment, our future research endeavors will extend beyond refining the methodology of 3D reconstruction. In addition to methodological advancements, we aim to harness the visual representations derived from MRI images of uterine myomas for widespread clinical applications. We intend to explore advanced algorithms, machine learning techniques, or innovative image processing methodologies that could further elevate the quality of our 3D models.

Our goal is to bridge the gap between research and practical clinical applications. To achieve this, we plan to collaborate with healthcare institutions and practitioners to implement our 3D uterine myoma models in various clinical scenarios.

This integration will encompass a wide range of applications, including but not limited to:

Preoperative Planning: We aim to provide surgeons with comprehensive 3D models that assist in surgical planning and decision-making. This includes the precise localization of myomas, estimation of surgical complexities, and selection of optimal incision sites.

Intraoperative Guidance: We will explore the real-time use of 3D models during surgeries, particularly in scenarios such as cesarean sections and myomectomy procedures, to aid surgeons in navigating complex anatomical structures.

Patient Education: We intend to develop educational tools that utilize 3D models to communicate with patients, helping them understand their condition and the proposed surgical interventions.

In summary, our forthcoming research endeavors will encompass a multifaceted approach, encompassing methodological enhancements, clinical integration, and rigorous validation. We aspire to translate our current achievements into tangible benefits for both patients and healthcare providers in the realm of uterine myoma diagnosis and treatment.

## 5 Conclusion

This study, applying an AI-powered uterine myoma diagnosis algorithm created by our team based on MRI, revealed a promising prospect on improving the efficiency of hysteroscopic myomectomy. The further stage needs more patients to refine the diagnosis algorithm and realize the achievements in clinical widely application.

## Data availability statement

The original contributions presented in the study are included in the article/**Supplementary Material**. Further inquiries can be directed to the corresponding authors.

## Ethics statement

The studies involving humans were approved by the scientific research ethics committee of Beijing Shijitan Hospital, Capital Medical University. The studies were conducted in accordance with the local legislation and institutional requirements. Written informed consent for participation was not required from the participants or the participants’ legal guardians/next of kin in accordance with the national legislation and institutional requirements.

## Author contributions

MC: Data curation, Investigation, Writing – original draft, Writing – review & editing. WK: Data curation, Formal analysis, Writing – review & editing. BL: Methodology, Resources, Writing – review & editing. ZT: Data curation, Resources, Writing – review & editing. CY: Data curation, Writing – review & editing. MZ: Data curation, Software, Writing – review & editing. HP: Software, Validation, Visualization, Writing – review & editing. WB: Funding acquisition, Project administration, Resources, Supervision, Validation, Writing – original draft, Writing – review & editing.
